# Aberrant KIF23 expression is associated with adverse clinical outcome and promotes cellular malignant behavior through the Wnt/β-catenin signaling pathway in Colorectal Cancer

**DOI:** 10.7150/jca.51565

**Published:** 2021-02-02

**Authors:** Zhiyu Ji, Aoning Mi, Mengmeng Li, Quanying Li, Changjiang Qin

**Affiliations:** Department of General Surgery, Huaihe Hospital of Henan University, Kaifeng, China.

**Keywords:** KIF23, Colorectal cancer, Proliferation, Migration, Wnt/β-catenin

## Abstract

**Purpose:** The aim of the present study was to reveal the clinicopathological significance and prognostic role of kinesin family member 23 (KIF23) in colorectal cancer (CRC) and characterize its biological function and the underlying mechanisms.

**Methods:** Bioinformatics analysis, immunohistochemistry, Western blot and qRT-PCR were utilized to investigate the expression of KIF23 in CRC tissues. The CCK-8 assay, wound healing assay and Matrigel assay were used to detect cell proliferation, migration and invasion *in vitro*. Western blot, immunofluorescence staining and cell function experiment were performed to explore the underlying mechanism.

**Results:** The overexpression of KIF23 was associated with T stage, N stage, M stage and TNM stage, and CRC patients with high KIF23 expression had a worse prognosis. KIF23 knockdown inhibits CRC cells proliferation, migration and invasion *in vitro*. The mechanism study determined that KIF23 activates the Wnt/β-catenin signaling pathway by promoting the nuclear translocation of β-catenin to regulate the malignant behavior of CRC cells.

**Conclusion:** These results suggest that KIF23 may act as a putative oncogene and a potential therapeutic target in CRC.

## Introduction

Colorectal cancer (CRC) is one of the most common malignant tumors of the digestive tract. Global cancer statistics reveal that the incidence of colorectal cancer ranks fourth in the world, with ~1850,000 new cases diagnosed annually 1. Related pathogenic factors of CRC have become increasingly explored in recent years, which to some extent prevent the incidence of CRC 2. However, the prognosis of CRC is still not optimistic 3. Therefore, it is of great significance to seek effective targets for diagnosing and treating CRC.

Kinesin family member 23 (KIF23), also known as MKLP1, is the human homolog of mouse KIF23 that is mainly located in the cytoplasm and nucleus and widely participates in cell mitosis by forming the spindle midbody 4,5. Previous studies have shown that KIF23 is a key regulator of mitosis 6. The abnormal function of KIF23 leads to a mitosis arrest and the appearance of binucleate or multinucleated cells, which leads to the occurrence of tumors 7,8. Some research shows that KIF23 is upregulated in a variety of tumors and is related to the occurrence and development of tumors, such as gastric cancer 9, glioma 10, breast cancer 11 and ovarian cancer 12. Zou and colleagues verified that overexpression of KIF23 was significantly associated with prognosis in ER-positive breast cancer patients and that knockdown of KIF23 inhibited tamoxifen-sensitive and resistant breast cancer cell proliferation and induced apoptosis 13. Therefore, KIF23 can be used as a tumor-associated antigen, and its overexpression is widely involved in tumor progression. However, the role of KIF23 in CRC has not been reported.

This study is the first to explore the clinical characteristics of KIF23 upregulation in colorectal cancer patients and its involvement in the malignant behavior of CRC cells through Wnt/β-catenin signaling. The results can provide new ideas for the diagnosis and targeted treatment of CRC.

## Material and methods

### Bioinformatics analysis

The RNASeq data for CRC were accessed from The Cancer Genome Atlas (TCGA, https://genome-cancer.ucsc.edu/) and the colorectal cancer cohort study came from Oncomine database (https://www.oncomine.org/). Kyoto encyclopedia of genes and genomes (KEGG) pathway analysis was used to find out the enriched pathways. The data were log2 transformed and analyzed using Excel 2009 and GraphPad Prism 5.0 software.

### Patients and tissue specimens

All paraffin samples were obtained from patients with primary CRC who underwent gastrointestinal surgery between February 2010 and February 2013 at the Huaihe Hospital of Henan University. The diagnosis of CRC was confirmed by at least two pathologists. Tumor staging was determined according to the eighth edition of the American Joint Committee on Cancer (AJCC) guidelines. Inclusion criteria were: Patients diagnosed with CRC by pathological diagnosis; patients have not undergone chemotherapy, radiotherapy or targeted therapy before surgery. Exclusion criteria were: Patients combined with other tumors; patients with incomplete clinical data; patients with poor compliance. Follow-ups were terminated by December 2019. Fresh colorectal cancer tissues and adjacent normal tissues were obtained from patients with colorectal cancer who underwent gastrointestinal surgery at Huaihe Hospital of Henan University. All tissues used for the study were immediately placed in liquid nitrogen after resection and then stored at -80 °C. The study was approved by the Ethics Review Committee of Huaihe Hospital of Henan University, and written informed consent was obtained from each patient.

### Immunohistochemical staining

The protocol of IHC staining was as previously described 14. Paraffin-embedded tissue specimens were sectioned continuously with a thickness of approximately 4 μM. Then, the sections were dewaxed with xylene and dehydrated by gradient ethanol. Then, the tissue sections were placed in a citric acid antigen retrieval buffer (pH 6.0) for antigen retrieval in a microwave oven. Endogenous peroxidase activity was inhibited by using 3% hydrogen peroxide for 10 min. Then, 3% BSA was added to the slide to cover the tissue evenly, and the slide was then sealed at room temperature for 30 min. The slide was incubated with rabbit polyclonal anti-KIF23 antibody overnight at 4 °C. On the following day, the secondary antibody was added and incubated at room temperature for 50 min. The slides were visualized with DAB and counterstained with hematoxylin. Finally, protein staining was observed under a microscope.

The degree of KIF23-positive staining was graded by semiquantitative microscopy 15. Sections were considered KIF23 positive when more than 10% of cells were stained. The staining intensity was scored as follows: 0, no staining; 1, weak staining; 2, moderate staining; and 3, strong staining. Negative or weak staining was categorized as low KIF23 expression, while moderate or strong staining was categorized as high KIF23 expression. Immunohistochemical staining results of tissue sections were scored by two pathologists who were blinded to the clinical and pathological features of the specimens.

### Cell culture and small interfering RNA transfection

Human colorectal cancer cell lines (HCT116, Caco2, SW480, LS174T, LOVO) and human normal colon epithelial cells (NCM460) were purchased from ATCC (American Type Culture Collection, Manassas, VA, USA). The cells were cultured in DMEM (DMEM, Biological Industries, USA) supplemented with 10% FBS and 1% penicillin/streptomycin. All cells were cultured in a humidified air incubator containing 5% CO_2_ at 37 °C.

Small interfering RNAs (siRNAs) targeting KIF23 (siKIF23#1: 5'- GGTAGATCTTGCTGGAAGT-3′; siKIF23#2: 5'- GGCTATTGTTACCGAACCT-3′; siKIF23#3: 5'- CAGTCACTAATGACGCTAA-3′) and a negative control (NC) RNA were synthesized by RiboBio (Guangzhou, China). Cells were seeded in six-well plates until 70% of the plate was covered and then transfected with siRNA using Lipofectamine^TM^ 3000 (Invitrogen, Carlsbad, CA, USA) according to the manufacturer's instructions. The transfection efficiency of siRNA was detected by qRT-PCR and Western blot.

### Western blot

The proteins were separated by SDS-PAGE gels and transferred to PVDF membranes (Millipore, Billerica, MA, USA). The membranes were blocked in TBST with 5% skimmed milk at room temperature for 1 h and incubated with primary antibodies overnight at 4 °C followed by incubation with rabbit secondary antibodies for 1 h. Antibody binding was detected on autoradiographic film using the enhanced chemiluminescence reagent (CWBIO, Beijing, China).

### qRT-PCR

Total RNA was isolated using TRIzol reagent (Servicebio, Boston, Massachusetts, USA). Complementary DNA (cDNA) was extracted by using the RevertAid First Strand cDNA Synthesis Kit (Thermo Fisher Scientific, Waltham, USA). The total RNA was determined by quantitative real-time PCR using FastStart Universal SYBR Green Master (Rox) (Roche, Switzerland).

### Proliferation assay

The cell proliferation assay was performed by using a Cell Counting Kit-8 (CCK-8, Dojindo Molecular Technologies, Rockville, MD, USA) reagent. CRC cells (100 μL) at a density of 3.0 × 10^5^ cells/well were added to the 96-well plate. Ten microliters of CCK-8 solution was added to each well at 0 h, 24 h, 48 h, 72 h and 96 h and cultured at 37 °C for an additional 4 h. The absorbance at a wavelength of 450 nm was measured using a microplate reader.

### Wound healing assay

The transfected cells were seeded into 6-well plates at a density of 3×10^5^ cells/mL and cultured to 80% confluence. Then, a linear wound was generated by scraping the cells with a sterile 10 μl pipette tip. Serum-free medium was added to each well after washing with PBS. The migration ability was evaluated by measuring changes in the size of the wound width or area. Images were captured after 0 h and 24 h in three fields.

### Matrigel assays

Cell invasion was measured in 24-well plates using Transwell chambers (8-μm pores, Corning, USA) coated with Matrigel (BD Biosciences, San Jose, CA, USA). Briefly, serum-free medium containing 3 × 10^5^ cells was added to the upper chamber of each well, and medium containing 10% fetal bovine serum was added to the lower chamber. After incubation at 37 °C and 5% CO_2_ for 24 h, the cells in the upper chamber were gently wiped off with cotton swabs. Then, 4% paraformaldehyde and 0.1% crystal violet were used to fix and stain the cells. Five visual fields were randomly selected to take images under a microscope.

### Immunofluorescence (IF) staining

HCT116 and SW480 cells were seeded onto coverslips in six-well plates, washed with phosphate buffered saline (PBS), fixed with 4% paraformaldehyde, and 0.5% Triton X-100 permeated for 10 minutes. After blocking with 5 % bovine serum albumin, the cells were cultured with primary antibody overnight at 4 °C. The cells were washed with PBS for 3 times and incubated with the secondary antibody for 1 hour at room temperature. The slides were subsequently stained with DAPI at room temperature for 5 min and visualized using a fluorescence microscope.

### Statistical analysis

SPSS version 20.0 (IBM, USA) was used for the analyses. The relationships between KIF23 expression and clinicopathological features were analyzed using χ^2^ tests. The survival curves were generated using Kaplan-Meier survival analysis and analyzed by the log-rank test. Univariate and multivariate Cox regression analyses were performed. Pearson's correlation analysis was used for revealing correlations. All differences were considered to be statistically significant at *P*<0.05.

## Results

### Expression of KIF23 in CRC tissues

**Figure 1:**
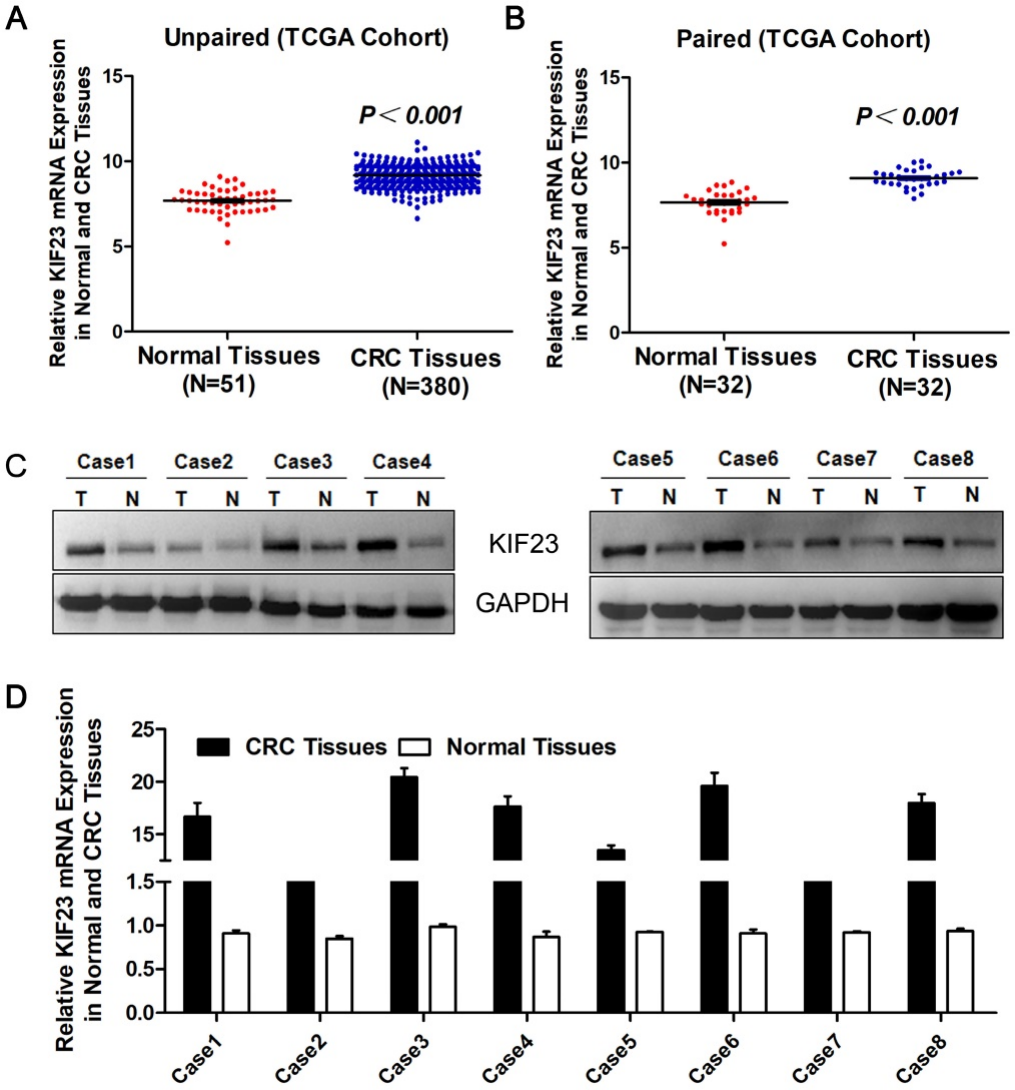
** Increase of KIF23 expression in CRC.** (A) Analysis of KIF23 expression in unpaired CRC (N = 380) and normal tissues (N = 51) in TCGA cohort (*P* < 0.001). (B) KIF23 expression in paired normal and CRC tissues (N = 32) in TCGA cohort (*P* < 0.001). (C) KIF23 protein levels were measured in CRC tissues and respective adjacent non-tumor tissues in 8 typical patients by Western blot. (D) Expression of KIF23 was analyzed by qRT-PCR in paired tumor and normal tissues.

To evaluate the expression level of KIF23 in CRC, we first studied the expression of KIF23 in TCGA cohort datasets. The results suggested that the levels of KIF23 mRNA in unpaired CRC tumors (N=380) were significantly upregulated compared with those in normal colorectal tissues (N=51) (*P*<0.001, Fig. 1A), and the upregulation of KIF23 in colorectal cancer was further confirmed in paired tumors and adjacent normal tissues (N=32, *P*<0.001, Fig. 1B). The same trend was observed in the colorectal cancer cohort from the Oncomine database (Table 1) 16-24. In addition, Western blot showed that KIF23 protein was upregulated in 8 pairs of fresh tissues obtained from our translational medical center (Fig. 1C). Similarly, KIF23 mRNA was significantly upregulated in colorectal cancer compared with paired normal colon tissues (N = 8, Fig. 1D).

**Figure 2:**
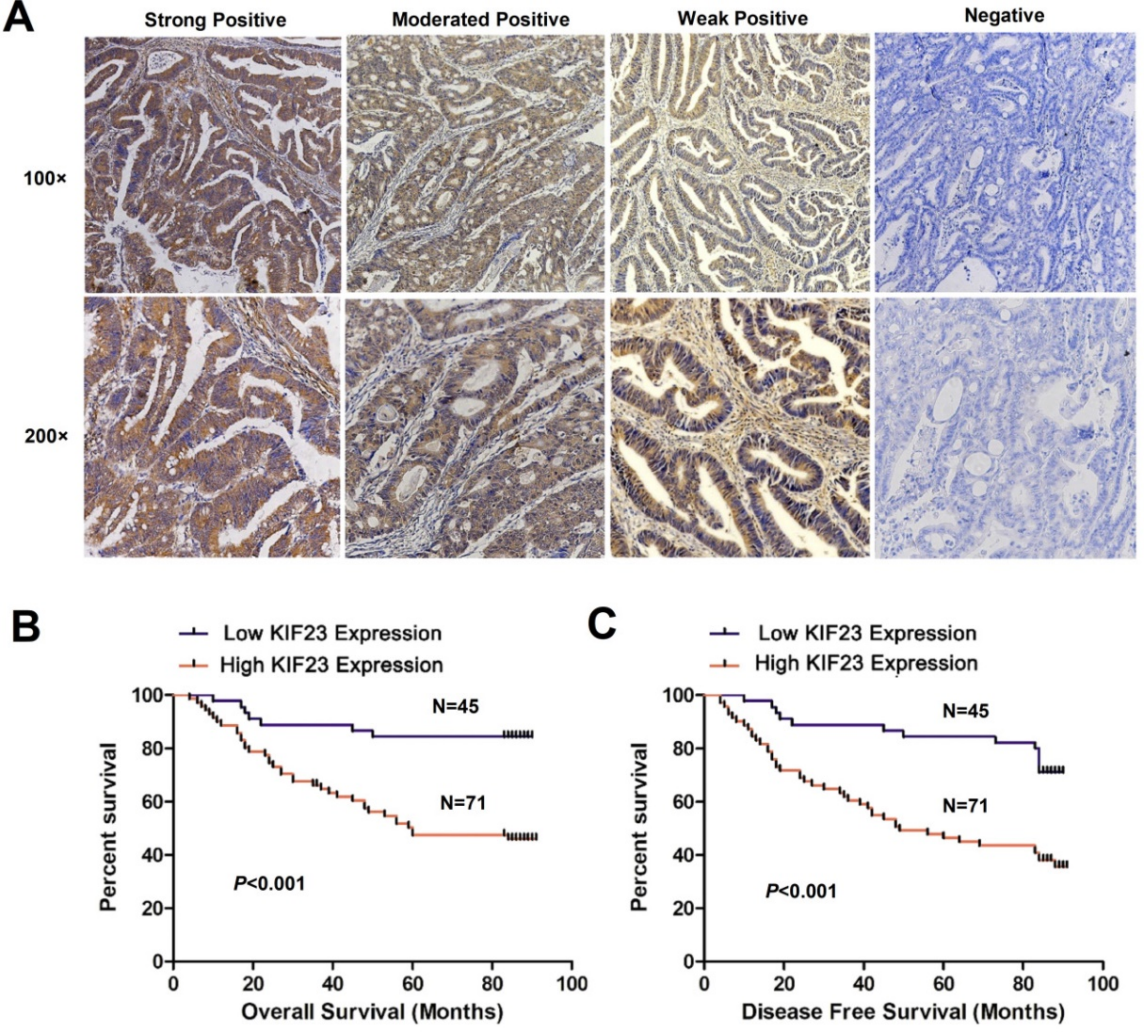
** Increased KIF23 protein expression predicts poor prognosis in patients with CRC.** (A) IHC staining of KIF23 protein in CRC tissues. (B and C) Patients with higher expression of KIF23 had a worse OS and DFS than those with lower KIF23 expression (*P* < 0.001).

To assess whether KIF23 is related to the clinical features of CRC patients, we performed immunohistochemistry on 116 colorectal cancer tissue specimens and scored the expression of KIF23. Representative KIF23 IHC staining images are presented in Fig. 2A, and KIF23-positive staining signals were mainly distributed in the cytoplasm. Overall, 71 patients (61.2%) had significantly higher expression in CRC tissues, and 45 patients (38.8%) had lower expression or negative expression (*P*<0.05).

Through the χ^2^ test, we obtained the correlation between KIF23 expression and the main clinical pathological features. As shown in Table 2, high KIF23 expression was closely related to T stage (*P*=0.008), N stage (*P*<0.001), M stage (*P*=0.013), and TNM stage (*P*=0.001). However, no statistically significant relationships were found between KIF23 expression and gender (*P*=0.122), age (*P*=0.763), tumor size (*P*=0.627), differentiation degree (*P*=0.520) or tumor location (*P*=0.919).

Next, we evaluated the relationship between KIF23 expression and the prognosis of CRC patients. By using Kaplan-Meier curves to compare the survival rates of the two groups, we found that high KIF23 expression was associated with worse overall survival (OS) and disease-free survival (DFS) than low KIF23 expression (*P*<0.001, Fig. 2B and 2C).

To determine whether KIF23 expression is an independent prognostic indicator of CRC, we performed univariate and multivariate analyses. The results are shown in Table 3. Univariate analyses suggested that tumor size (HR=2.093, 95% CI: 1.136-3.857, *P*=0.018), differentiation degree (HR=3.520, 95% CI: 1.954-6.339, *P*<0.001), T stage (HR=5.434, 95% CI: 2.822-10.464, *P*<0.001), N stage (HR=2.298, 95% CI: 1.616-3.267, *P*<0.001), M stage (HR=4.564, 95% CI: 2.374-8.776, *P*<0.001), TNM stage (HR=2.608, 95% CI: 1.786-3.809, *P*<0.001), and KIF23 expression (HR=4.394, 95% CI: 1.959-9.855, *P*<0.001) were significantly associated with OS. The multivariate analysis demonstrated that high KIF23 expression was an independent prognostic predictor of CRC patients (HR=3.351, 95% CI: 1.341-8.373, *P*=0.010). In addition, KIF23 expression was also an independent predictor of DFS (HR=2.889, 95% CI: 1.409-5.924, *P*=0.004) in CRC patients (Table 4).

### Expression of KIF23 in CRC cell lines

**Figure 3:**
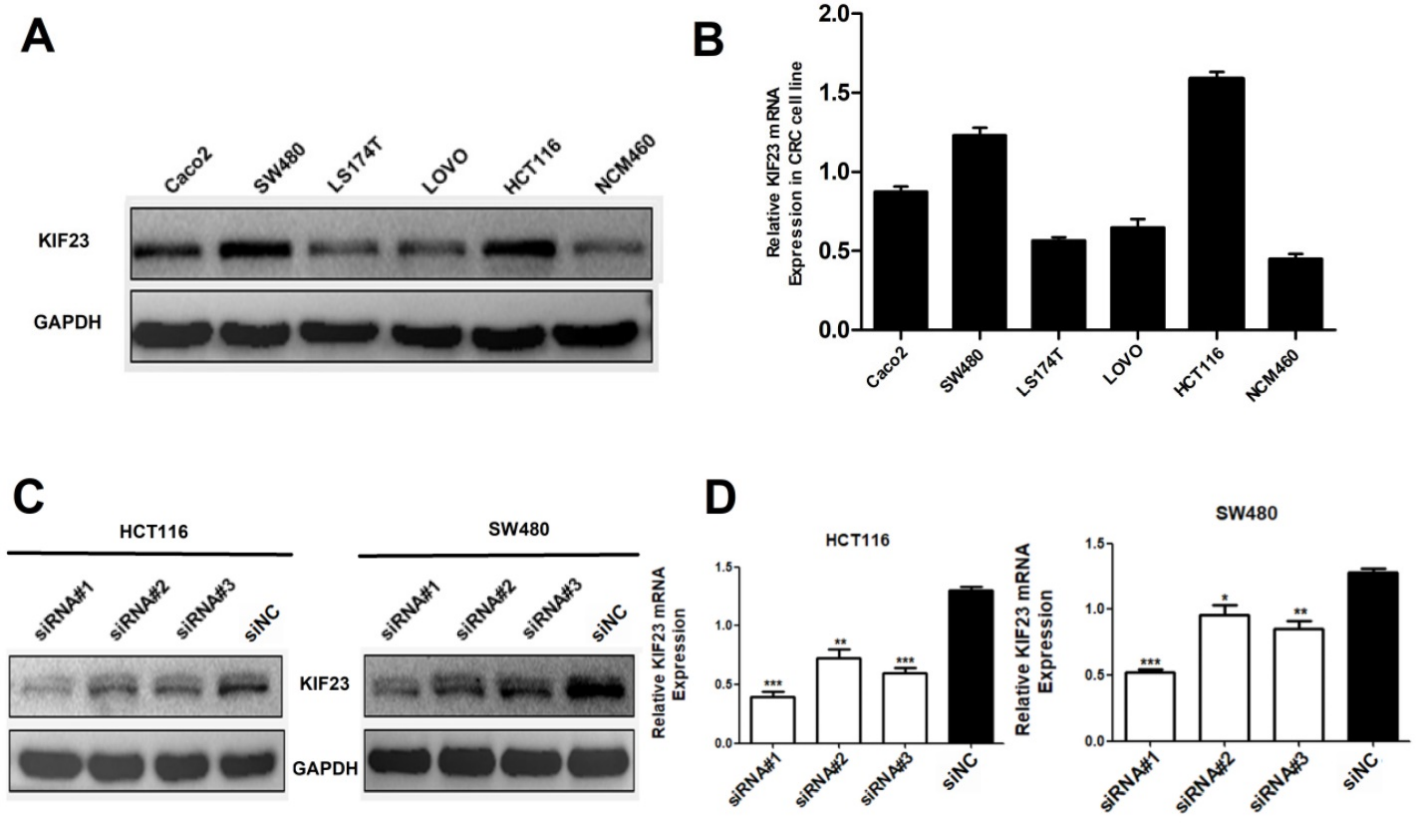
** Expression of KIF23 in CRC cell lines.** (A and B) the KIF23 protein and mRNA levels in six cell lines was examined by Western blot assays and qRT-PCR. (C and D) The siRNA transfection efficiency was detected by Western blot assays and qRT-PCR. *P < 0.05; **P < 0.01; ***P < 0.001.

Considering that high expression of KIF23 is significantly related to the poor prognosis of patients, we then studied the biological function of KIF23 in CRC cells. By qRT-PCR and Western blot analysis of KIF23 mRNA and protein expression in six cell lines, we found that HCT116 and SW480 cells had higher expression of KIF23 (Fig. 3A and 3B). Therefore, these two cell lines were selected for subsequent research. To further examine the effect of KIF23 on the biological function of CRC, we used siRNA transfection technology to silence the expression of KIF23 in HCT116 and SW480 cell lines and detected the transfection efficiency by Western blot analysis and qRT-PCR (Fig. 3C and 3D).

### KIF23 promotes CRC cell proliferation, migration and invasion *in vitro*

**Figure 4:**
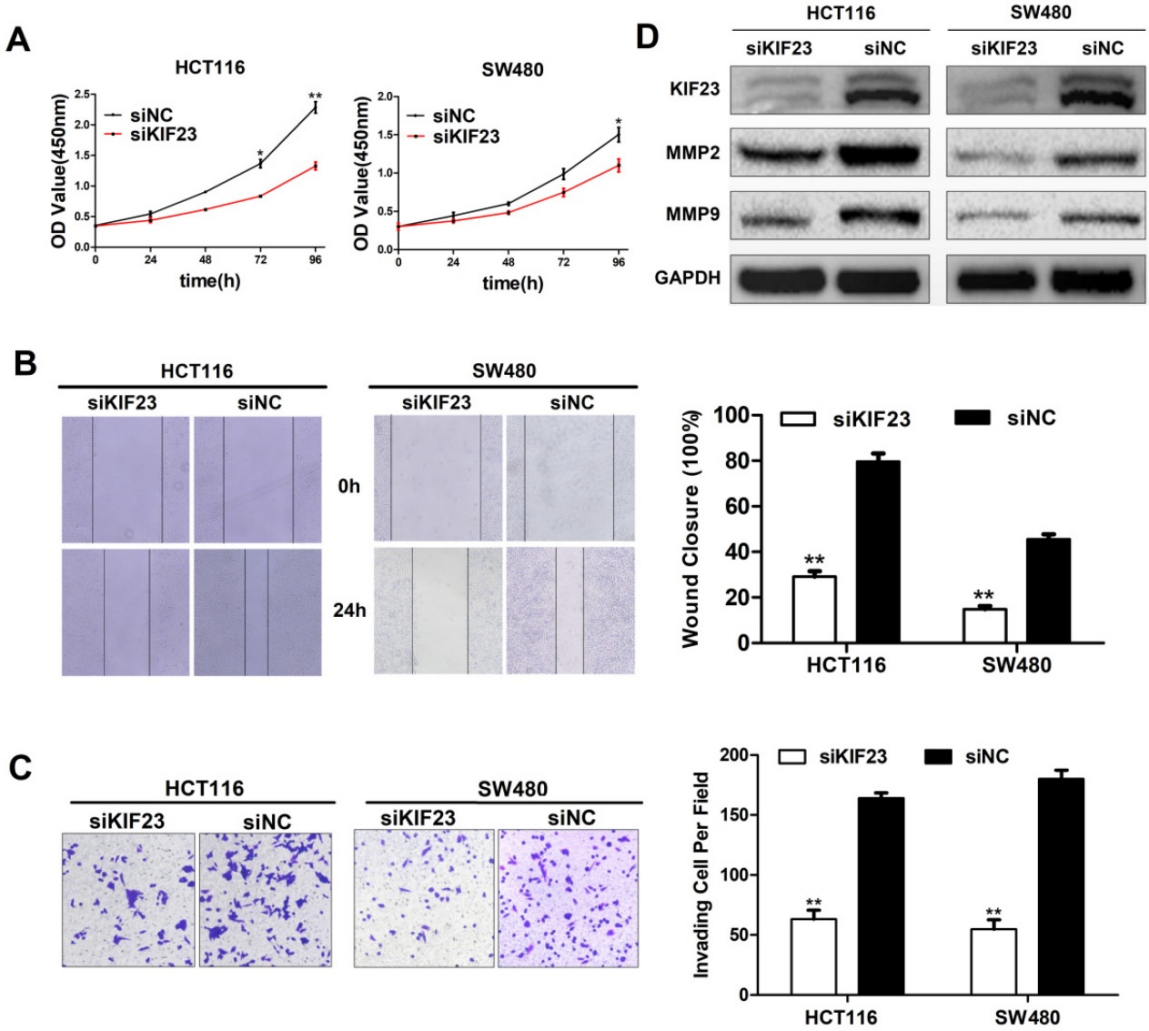
** Effect of KIF23 on CRC cell proliferation, migration and invasion.** (A) Cell proliferation activity was assessed by CCK-8 assay. (B) Wound healing assays were performed to investigate the role of KIF23 in HCT116 and SW480 cell migration. (C) Matrigel assays were used to detect the role of KIF23 in cell invasion. (D) The protein expression of MMP2 and MMP9 in different transfected groups was determined by Western blot. *P < 0.05; **P < 0.01.

As a motility-related protein, KIF23 plays an important role in the mitosis and motility of cells. We chose the two CRC cell lines (HCT116 and SW480) with the highest expression level of KIF23 and constructed KIF23-silenced cell lines via siRNA transfection. CCK-8 assays suggested that KIF23 knockdown greatly reduced the proliferation ability of CRC cells (Fig. 4A). The wound healing assay indicated that the migration ability of si-KIF23 cells was significantly decreased compared to that of control cells (Fig. 4B). Next, we examined the role of KIF23 in tumor cell invasion. Matrigel Transwell assays showed that lower KIF23 expression significantly reduced the invasion rate (Fig. 4C).

Based on the fact that MMPs (matrix metalloproteinases) are related to cell migration and invasion, we detected the expression of MMP2 and MMP9 in KIF23 silenced cells by Western blot analysis. The results showed that the protein levels of MMP2 and MMP9 were significantly decreased in KIF23 silencing group (Fig. 4D), which was consistent with the migration and invasion results of HCT116 and SW480 cells with different KIF23 expression levels.

### KIF23 mediates malignant behavior in CRC via the Wnt/β-catenin signaling pathway

**Figure 5:**
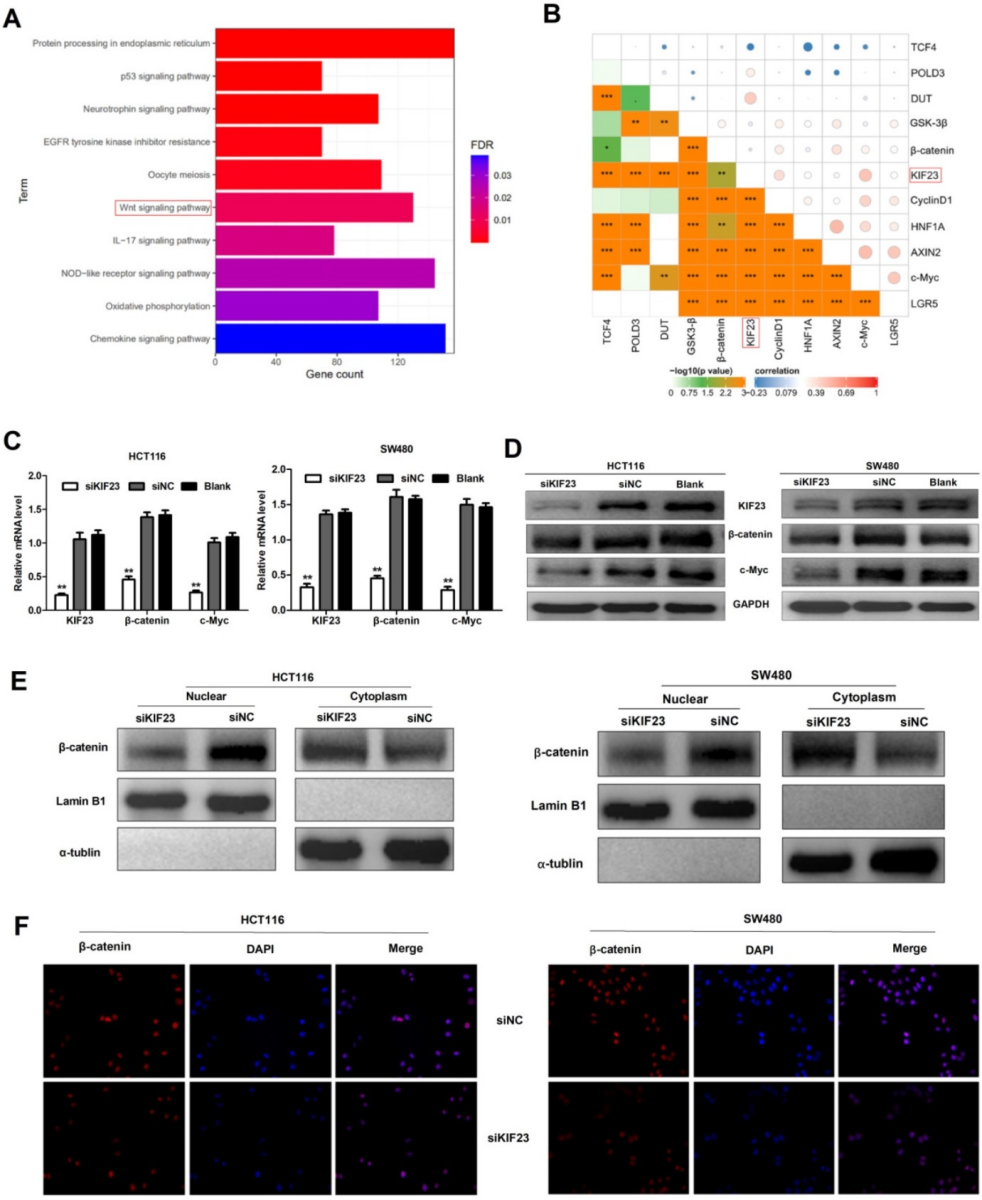
** KIF23 regulates Wnt/β-catenin signaling in CRC.** (A) Correlation between KIF23 and Wnt pathway by KEGG pathway enrichment analysis. (B) Pearson's correlation analysis was used to evaluate the correlation between KIF23 and Wnt targets. (C) The mRNA expression of KIF23, β-catenin and c-Myc in different transfected groups was determined by qRT-PCR. (D) The protein expression of KIF23, β-catenin and c-Myc in different transfected groups was determined by Western blot. (E) The level of β-catenin in cell nucleus and cytoplasm was determined by Western blot. (F) β-catenin nuclear translocation was detected by IF staining. *P < 0.05; **P < 0.01; ***P < 0.001.

Previous research reported that Wnt/β-catenin signaling plays an important role in CRC 25. To investigate whether Wnt/β-catenin signaling pathway is involved in KIF23-mediated CRC progression, a series of assays were conducted to assess the association between Wnt/β-catenin signaling and KIF23. Firstly, we found that KIF23 was related to Wnt pathway through KEGG pathway enrichment analysis (Fig. 5A). Then we analyzed the correlation between KIF23 and 10 reported Wnt targets in TCGA database. The results showed that KIF23 was positively correlated with most targets, including β-catenin, c-Myc, GSK3-β, CyclinD1, DUT, LGR5, AXIN2 and POLD3 (P < 0.001, R > 0.2, Fig. 5B). qRT-PCR analysis and Western blot also confirmed these results that KIF23 knockdown could significantly reduce the expression levels of β-catenin and c-Myc (Fig. 5C and 5D). Moreover, subcellular protein fraction study confirmed that KIF23 knockout decreased the level of β-catenin in nucleus and increased the level of β-catenin in cytoplasm (Figure ​5E). To confirm β-catenin translocation, we performed immunofluorescence staining and found that β-catenin was decreased in nucleus of CRC cells with silencing of KIF23 (Figure 5F). These data indicated that KIF23 promotes the nuclear translocation of β-catenin in CRC cells to regulate Wnt/β-catenin signaling pathway.

**Figure 6:**
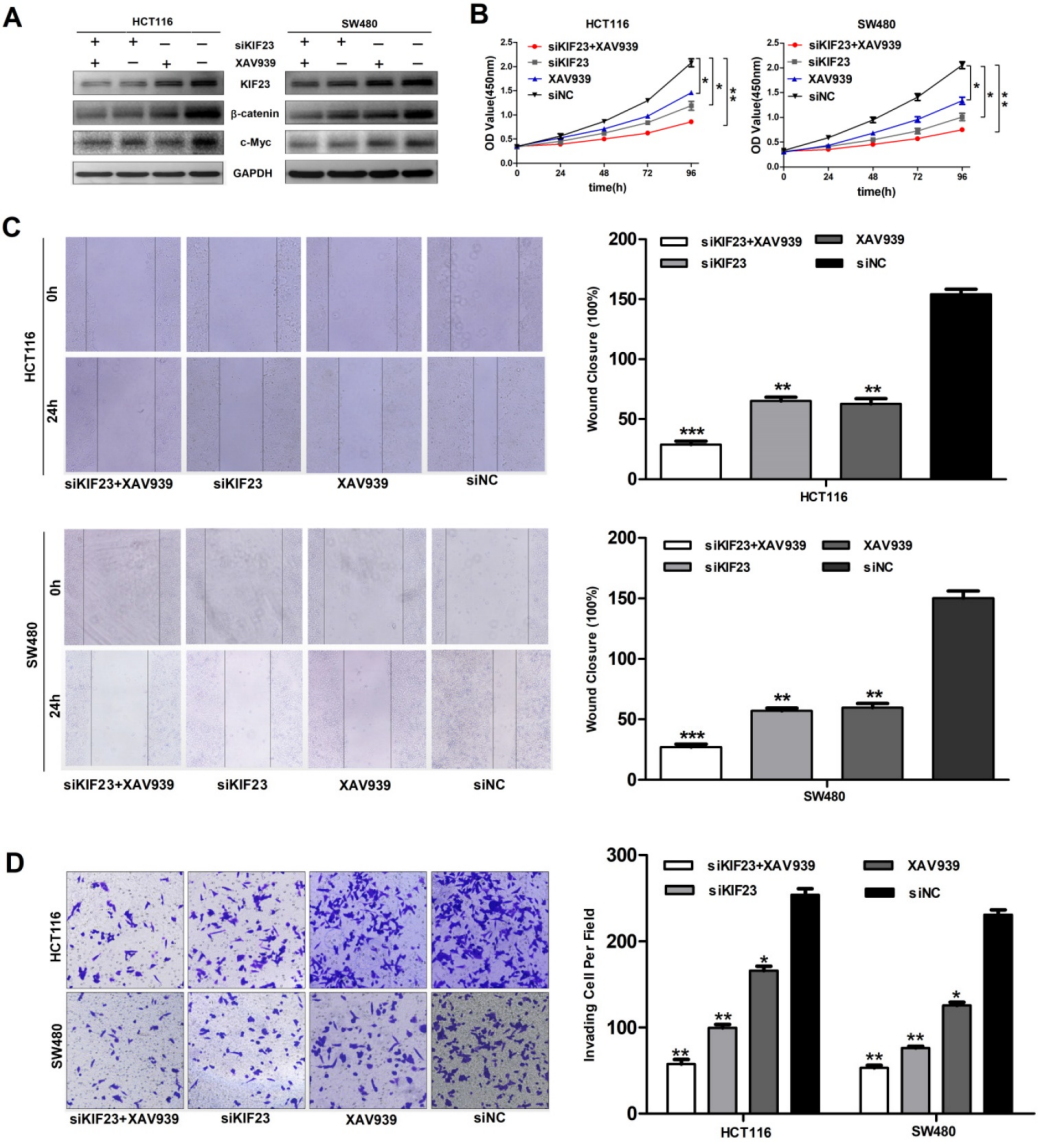
** KIF23 mediates malignant behaviors in CRC via the Wnt/β-catenin pathway.** (A) Western blot analysis of expression of β-catenin and c-Myc in different transfected groups with or without the administration of Wnt/β-catenin pathway, XAV939. (B) The proliferation of HCT116 and SW480 cells with KIF23 siRNA or negative control transfection cultured in medium with or without XAV939; (C) The migration of HCT116 and SW480 cells with KIF23 siRNA or negative control transfection cultured in medium with or without XAV939; (D) The invasion of HCT116 and SW480 cells with KIF23 siRNA or negative control transfection cultured in medium with or without XAV939; *P < 0.05; **P < 0.01; ***P < 0.001.

Subsequently, we explored whether direct interference in the Wnt pathway could affect the function of CRC cell lines. We found that the additional administration of the Wnt/β-catenin pathway inhibitor XAV939 further inhibited the expression of β-catenin and c-Myc (Fig. 6A). Moreover, Wnt pathway inhibitors XAV939 and KIF23 silencing can inhibit the proliferation, migration and invasion of CRC cells and the inhibitory effect is significantly enhanced when the two work together (Fig. 6B, 6C and 6D). Taken together, these data indicated that KIF23 silencing inhibited the progression of CRC through the Wnt/β-catenin signaling pathway.

## Discussion

Early diagnosis and surgery are key to the treatment of CRC. In some studies, only 2% of all CRC patients were diagnosed early 26. When cancer is in the middle and late stages, chemotherapy and immunotherapy can provide the most benefits to patients. At present, new immune-based treatment methods for CRC are being developed 27, and many new targets for CRC treatment have been identified, such as KIF20A and miRNA-875-3p 28,29. In our study, we demonstrated the relationship between KIF23 and CRC, which can be used as a new target for CRC treatment. Of course, its therapeutic effect needs to be verified.

KIF23 is recruited by the chromosomal passenger protein into the spindle midzone/midbody to form the midbody and complete cell division in human cells 7. Downregulating the expression of KIF23 inhibits the formation of mesosomes and cytokinesis, which results in cell division defects and the formation of multinucleated cells, ultimately leading to cancer 7,30. Several studies have shown that KIF23 plays a crucial role in cancer. Li et al. showed that KIF23 is associated with poor prognosis in ovarian cancer and promotes proliferation, migration, and cell cycle progression 12. Satoshi Takahashi's study showed that silencing KIF23 in glioma can inhibit the proliferation of glioma cells both *in vitro* and *in vivo* 10. In addition, KIF23 and KIF20A, as members of the kinesin-6 subfamily, have similar functions in maintaining normal cell division 31. Our previous studies have shown that high expression of KIF20A can significantly promote the proliferation of colorectal cancer, and it is related to the poor prognosis of colorectal cancer patients 32. Therefore, we speculate that KIF23 may play a similar role as KIF20A in colorectal cancer.

In this study, we found that the mRNA and protein levels of KIF23 in CRC were higher than those in normal colorectal tissues. Through clinicopathological analysis, we also found that patients with high expression of KIF23 had worse prognosis than those with low expression of KIF23. This is consistent with our previous speculation. In addition, Cox regression analysis showed that the expression level of KIF23 was an independent risk factor for the overall survival and disease-free survival of colorectal cancer patients. Therefore, we conclude that KIF23 may be a molecular marker to predict the development and prognosis of CRC.

Then, we explored the biological function of KIF23 in colorectal cancer. We used siRNA targeted detection to knock out KIF23 in CRC cells. The results showed that KIF23 promoted the malignant behavior of colorectal cancer by enhancing the proliferation, migration and invasion of CRC cells.

The Wnt/β-catenin pathway regulates cell migration, survival and self-renewal in embryonic and adult tissue homeostasis 33. Abnormal Wnt signaling is associated with the development of several pathologies, including cancer 34. A large amount of evidence shows that Wnt/β-catenin signaling plays an important role in the proliferation, migration, invasion and angiogenesis of cancer cells 35,36. In the Wnt signaling cascade, Wnt/β-catenin signaling is dysregulated in approximately 90% of colorectal cancer 3. These changes mainly come from the β-catenin gene, which is the main effector molecule of the Wnt/β-catenin pathway 37,38. Studies have shown that in the absence of Wnt ligands, the “degradation complex” formed by APC, axis inhibitor (Axin) and glycogen synthetase kinase-3-β (GSK3-β) phosphorylates β-catenin in cells, which is subsequently degraded by the ubiquitin-proteasome system 39. When Wnt protein binds to Frizzled (Fzd) and LDL receptor-related protein, GSK3-β in the cell is inactivated, and the formation of degradation complexes is inhibited, which causes β-catenin to accumulate in the cytoplasm and then transfer to the nucleus 40. In the nucleus, β-catenin interacts with TCF/LEF family proteins to form the β-catenin-TCF4 complex, which eventually activates a series of downstream genes related to cell proliferation and metastasis, such as c-Myc and cyclin D1 41,42. Moreover, some studies have found that KIF23 promotes the proliferation of gastric cancer cells through the Wnt/β-catenin signaling pathway 43. We observed the same phenomenon in colorectal cancer. Our results showed that silencing KIF23 inhibited the expression of β-catenin and c-Myc, and KIF23 regulates the malignant behavior of CRC cells by promoting the accumulation of β-catenin in the nucleus to activate the Wnt/β-catenin signaling pathway.

## Conclusion

Our study demonstrated that KIF23 overexpression predicts poor prognosis in colorectal cancer patients and promotes the proliferation, migration and invasion of colorectal cancer cells through the Wnt/β-catenin signaling pathway. Therefore, monitoring the expression of KIF23 may provide an important reference for clinical diagnosis, treatment and prognosis evaluation of colorectal cancer.

## Figures and Tables

**Table 1:** Oncomine analysis of KIF3 expression in CRC (total 11 CRC cohorts)

Cohort No.	Cohort	Sample (N)	t-test	Fold-change	*P*-value
1	Ki Colon 16	Colon Adenocarcinoma (50) vs. Normal (28)	9.492	2.556	2.99E-15
2	Skrzypczak Colorectal 2 17	Colon Adenoma (5) vs. Normal (10)	9.449	5.475	1.90E-7
		Colon Carcinoma (5) vs. Normal (10)	9.686	4.768	8.03E-7
3	Sabates-Bellver Colon 18	Rectal Adenoma (7) vs. Normal (32)	9.024	4.983	4.12E-6
		Colon Adenoma (25) vs. Normal (32)	9.041	3.683	1.05E-11
4	TCGA Colorectal	Cecum Adenocarcinoma (22) vs. Normal (22)	8.215	2.642	2.48E-10
		Colon Mucinous Adenocarcinoma (22) vs. Normal (22)	7.557	2.340	1.46E-9
		Rectal Adenocarcinoma (6) vs. Normal (22)	5.583	2.166	5.40E-5
5	Gaedcke Colorectal 19	Rectal Adenocarcinoma (65) vs. Normal (65)	10.221	2.171	1.35E-18
6	Skrzypczak Colorectal 20	Colorectal Carcinoma (36) vs. Normal (24)	5.649	2.423	5.36E-7
7	Alon Colon 17	Colon Adenocarcinoma (39) vs. Normal (22)	3.679	1.366	2.89E-4
8	Notterman Colon 21	Colon Adenocarcinoma (18) vs. Normal (18)	2.005	1.756	0.026
9	Gaspar Colon 22	Colorectal Adenoma Epithelia (56) vs. Normal (22)	2.231	1.226	0.015
10	Hong Colorectal 23	Colorectal Carcinoma (70) vs. Normal (12)	7.305	2.821	4.13E-7
11	Kaiser Colon 24	Rectosigmoid Adenocarcinoma (10) vs. Normal (5)	4.039	1.983	0.001
		Cecum Adenocarcinoma (17) vs. Normal (5)	4.394	2.008	7.65E-4
		Colon Adenocarcinoma (41) vs. Normal (5)	4.942	2.047	7.92E-4
		Colon Mucinous Adenocarcinoma (13) vs. Normal (5)	2.959	1.717	0.006
		Rectal Adenocarcinoma (8) vs. Normal (5)	1.818	1.471	0.048
		Rectal Mucinous Adenocarcinoma (4) vs. Normal (5)	1.625	1.310	0.074

**Table 2:** Correlation between KIF23 expression and clinicopathological variables in 116 CRC patients

Characteristics	Total	KIF23 expression		c^2^ Value	*P* Value
		Low (N= 45)	High (N= 71)		
**Gender**				2.391	0.122
Male	67	30	37		
Female	49	15	34		
**Age**				0.091	0.763
<60 years	51	19	32		
≥60 years	65	26	39		
**Tumor size**				0.237	0.627
<5 cm	56	23	33		
≥5 cm	60	22	38		
**Differentiation degree**				1.306	0.520
Well	3	1	2		
Moderate	86	31	55		
Poor	27	13	14		
**Tumor location**				0.941	0.919
Ascending colon	26	11	15		
Transverse colon	7	3	4		
Descending colon	4	1	3		
Sigmoid colon	23	10	13		
Rectum	56	20	36		
**T stage**				9.766	0.008
1	0	0	0		
2	13	9	4		
3	91	35	56		
4	12	1	11		
**N stage**				18.984	<0.001
0	60	34	26		
1	32	9	23		
2	24	2	22		
**M stage**				6.129	0.013
0	99	43	56		
1	17	2	15		
**TNM stage**				16.245	0.001
I	10	7	3		
II	47	25	22		
III	42	11	31		
IV	17	2	15		

**Table 3:** Cox proportional hazard regression analysis for overall survival

Characteristics	Univariate analysis				Multivariate analysis			
	HR	95% CI		*P* value	HR	95% CI		*P* value
		Lower	Upper			Lower	Upper	
Gender	1.402	0.781	2.515	0.258				
Age	0.675	0.376	1.212	0.188				
Tumor size	2.093	1.136	3.857	0.018				
Differentiation degree	3.520	1.954	6.339	<0.001	3.885	1.926	7.834	<0.001
Tumor location	1.082	0.897	1.304	0.412				
T stage	5.434	2.822	10.464	<0.001				
N stage	2.298	1.616	3.267	<0.001				
M stage	4.564	2.374	8.776	<0.001				
TNM stage	2.608	1.786	3.809	<0.001				
KIF23 expression	4.394	1.959	9.855	<0.001	3.351	1.341	8.373	0.010

Abbreviations: HR, hazard ratio; CI, confidence interval.

**Table 4:** Cox proportional hazard regression analysis for disease free survival

Characteristics	Univariate analysis				Multivariate analysis			
	HR	95% CI		*P* value	HR	95% CI		*P* value
		Lower	Upper			Lower	Upper	
Gender	1.320	0.788	2.212	0.291				
Age	0.773	0.462	1.296	0.329				
Tumor size	1.845	1.085	3.138	0.024				
Differentiation degree	2.541	1.479	4.367	0.001	2.617	1.369	5.002	0.004
Tumor location	1.092	0.925	1.289	0.299				
T stage	4.186	2.265	7.737	<0.001				
N stage	1.828	1.337	2.499	<0.001				
M stage	3.414	1.826	6.380	<0.001				
TNM stage	1.925	1.390	2.665	<0.001				
KIF23 expression	3.109	1.673	5.778	<0.001	2.889	1.409	5.924	0.004

Abbreviations: HR, hazard ratio; CI, confidence interval.
